# Sturzprävention bei älteren Menschen durch ergotherapeutische Wohnraumanalyse, -beratung und -anpassung: eine Prozessdarstellung

**DOI:** 10.1007/s00391-022-02103-w

**Published:** 2022-09-07

**Authors:** Sara Mohr, Christian Müller, Frank Hildebrand, Markus Laubach

**Affiliations:** 1 Amsterdam University of Applied Sciences, Amsterdam, Niederlande; 2 Ergo Unterwegs GbR, Bruchsal, Deutschland; 3 SHG-Bildung, Fachschule für Ergotherapie, Saarbrücken, Deutschland; 4 Klinik für Orthopädie, Unfall- und Wiederherstellungschirurgie, Uniklinik RWTH Aachen, Aachen, Deutschland

**Keywords:** Osteoporose, Ergotherapie, Sturzrisiko, Screening, Gesundheitsförderung, Osteoporosis, Occupational therapy, Fall risk, Screening, Health promotion

## Abstract

Sturzgeschehen bei älteren Menschen treten häufig auf und sind mit relevanten kurz- als auch langfristigen Traumafolgen assoziiert. Vor dem Hintergrund der demografischen Entwicklung rücken präventive Maßnahmen zunehmend in den Fokus. Wohnraumanalysen durch ErgotherapeutInnen ermöglichen die Evaluation extrinsischer und verhaltensbasierter Sturzrisikofaktoren. Verhältnisprävention im Sinne der ergotherapeutischen Wohnraumanalyse ist eine wirksame Maßnahme, um Morbidität und Mortalität, einhergehend mit Stürzen bei älteren Menschen, und den steigenden sturzassoziierten Gesundheitskosten vorzubeugen. Ergotherapeutische Wohnraumanalysen und entsprechende Sturzpräventionsmaßnahmen reduzieren Sturzrisiko und Sturzrate bei älteren Menschen mit erhöhtem Sturzrisiko effektiv und sind im Kontext bestehender gesetzlicher Rahmenbedingungen umsetzbar. Ziel dieser Übersichtsarbeit ist es, den optimalen Versorgungsprozess der Wohnraumanalyse, -beratung und -anpassung für sturzgefährdete PatientInnen durch HausärztInnen, OrthopädInnen und ErgotherapeutInnen darzustellen.

**Zusatzmaterial online:**

Zusätzliche Informationen sind in der Online-Version dieses Artikels (10.1007/s00391-022-02103-w) enthalten.

## Abstract (en)

Falls occur frequently in old people and are associated with relevant short-term and long-term trauma sequelae. Particularly in the light of demographic change, preventive measures such as falls prevention are increasingly gaining in importance. Occupational therapy home assessments enable the evaluation of external and behavioral risk factors for falls in the context of environmental prevention. Environmental prevention is an effective measure in preventing morbidity and mortality related to falls in old people and the increase in healthcare costs associated with falls. Occupational therapy home assessments effectively reduce fall risk and fall rate among older people at risk of falling and can be implemented in the context of existing regulatory frameworks. The aim of this overview is to illustrate the optimal process of home assessment and adaptation for patients at risk of falling by general practitioners, orthopedic surgeons and occupational therapists.

## Einleitung

Stürze stellen bei älteren Menschen eine der führenden Ursachen für Verletzungen und Todesfälle dar. Pro Jahr erleiden ca. 30–40 % der Menschen über 65 Jahre einen Sturz, wobei ein hoher Anteil wiederholt stürzt [11]. Damit sind Stürze nicht nur der wichtigste Risikofaktor für Frakturen, sie führen häufig auch zu chronischen Schmerzsyndromen, Funktionseinschränkung, signifikanten Gesundheitskosten und erhöhter Sterblichkeit [18, 32]. Aus volkswirtschaftlicher Sicht bringt diese Entwicklung *ceteris paribus* eine Steigerung sturzassoziierter Diagnostik- und Behandlungskosten mit sich, was die Notwendigkeit der Implementierung evidenzbasierter Sturzprävention in den Fokus rückt.

## Hintergrund

**Figure 1:**
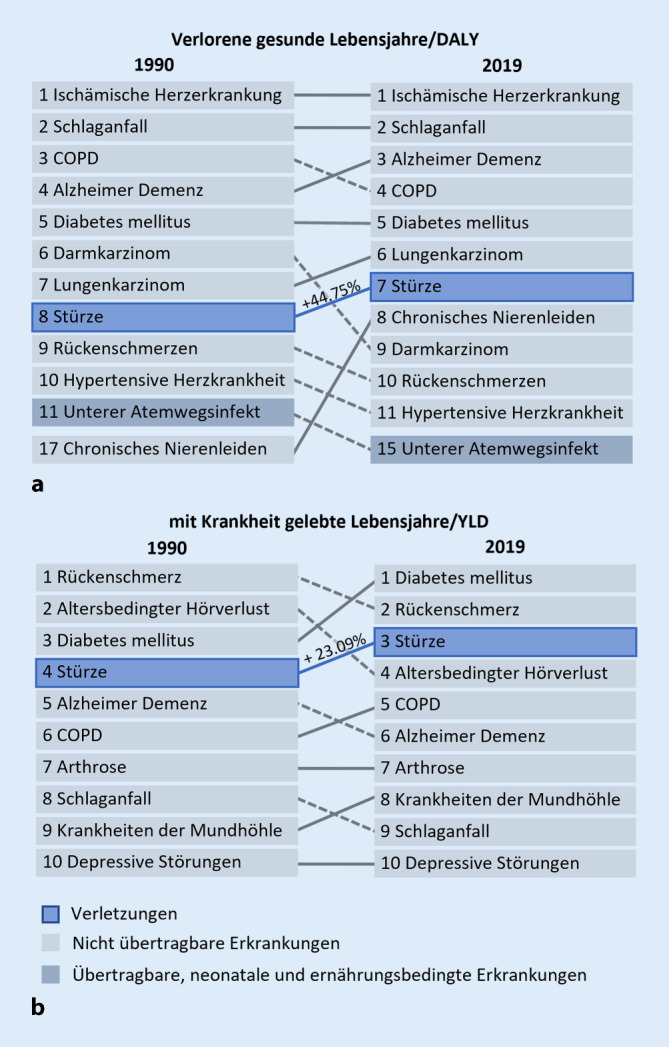

Die Bedeutung sturzpräventiver Maßnahmen zeigt sich angesichts von Prognosen, die bis 2030 von einer Verdoppelung der Sturzgeschehen ausgehen [20]. Für Hüftfrakturen, die zu über 90 % sturzassoziiert sind, wird erwartet, dass die Behandlungskosten von 2002 bis 2050 um 128 % steigen [20]. Stürze und Sturzangst führen zu einer Reduktion der Alltagsaktivitäten, was erneute Stürze begünstigt [23, 35]. Bereits jetzt verzeichnen die Anzahl der mit Krankheit gelebten Lebensjahre sowie der Verlust potenziell gesunder Lebensjahre aufgrund Sturzleiden in Deutschland in den letzten drei Jahrzehnten einen deutlichen Anstieg (Abb. 1).

Das individuelle Sturzrisiko setzt sich aus 3 Faktorengruppen zusammen [30]:

- intrinsische Faktoren, assoziiert mit der Person, z. B. Alter, Vorerkrankungen,
- extrinsische Faktoren, assoziiert mit der individuellen Umwelt, z. B. schlechte Beleuchtung, glatte Fußböden,
- verhaltensbasierte Faktoren, z. B. riskante Aktivitäten, mangelndes Gefahrenbewusstsein.

Die unmittelbare Sturzursache ist häufig eine Kombination aus Risikofaktoren und extrinsischen Auslösern [31]. Die meisten älteren Menschen stürzen im häuslichen Umfeld [34], wobei meist intrinsische Faktoren das Sturzrisiko erhöhen, während extrinsische Faktoren in über 30 % sturzauslösend sind [31]. Bestehende Präventionsmaßnahmen legen den Fokus auf Verhaltensprävention durch Kraft- und Gleichgewichtstraining, welches intrinsische Sturzrisikofaktoren adressiert [17]. Die Adhärenz der PatientInnen für eigenständige, langfristige Umsetzung von Trainingsprogrammen zur Verhaltensprävention ist jedoch gering [28], insbesondere da Betroffene häufig extrinsische Ursachen für Stürze verantwortlich machen [6, 27]. Aufgrund des multifaktoriellen Sturzgeschehens sollten im Rahmen der Prävention alle Sturzrisikofaktoren zur Identifikation von Risikogruppen und Auswahl passender Interventionen bedacht werden [11, 13, 14].

**Figure 2:**
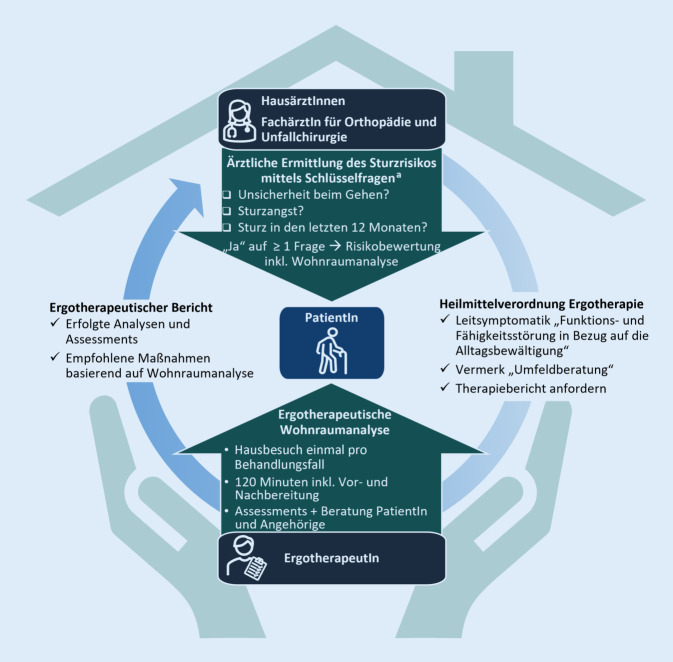

Zentraler Bestandteil einer Intervention im Bereich der verhaltensbasierten und extrinsischen Sturzrisikofaktoren ist das Assessment der Wohnumgebung. Das Tätigkeitsfeld der Ergotherapie erstreckt sich auf Maßnahmen der Wohnraumanalyse, -beratung und -anpassung, um die Sicherheit im häuslichen Umfeld zu erhöhen, Stürze zu verhindern, und Einschränkung der Aktivitäten des täglichen Lebens zu vermeiden [33]. In dieser Übersichtsarbeit wird der interdisziplinäre Prozess zur Sturzprävention bei älteren Menschen durch ergotherapeutische Wohnraumanalysen in Deutschland aufgezeigt (Abb. 2).

## Ärztliche Ermittlung des Sturzrisikos

Die Leitlinie der Deutschen Gesellschaft für Allgemeinmedizin und Familienmedizin empfiehlt das jährliche Befragen älterer PatientInnen nach zurückliegenden Stürzen als Teil des geriatrischen Assessments in der Hausarztpraxis [7]. Da eine verminderte Knochendichte signifikant mit einem erhöhten Sturzrisiko assoziiert ist, sollten insbesondere diese PatientInnen eine entsprechende Risikoanalyse erhalten [24]. Somit sind im Sinne der integrierten Versorgung neben HausärztInnen auch OrthopädInnen und UnfallchirurgInnen zentrale Anlaufstelle für die Sturzrisikoanalyse. Basis hierfür bildet das *Disease-Management-Programm* (DMP) Osteoporose [9, 10]. Dieses wurde am 01.07.2021 etabliert, und es wird mit einem Fortschreiten der Implementierung in naher Zukunft gerechnet [22].

**Figure 3:**
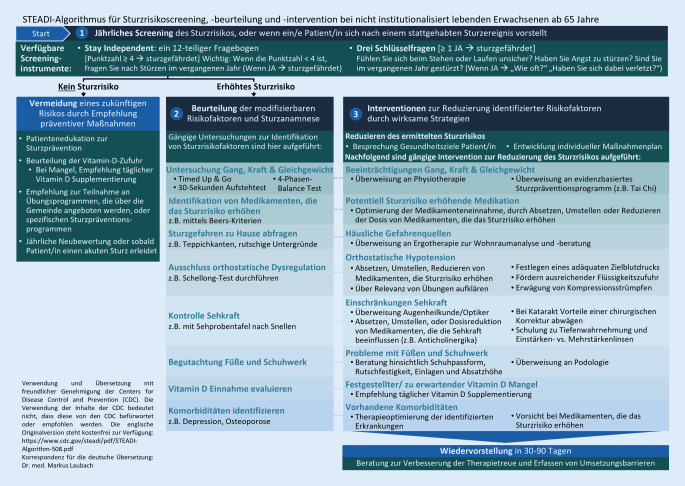

In Deutschland gibt es aktuell keine spezifischen Leitlinien zur Sturzprävention, welche validierte Assessments und Algorithmen bieten. Basierend auf den Leitlinien zur Sturzprävention der amerikanischen und britischen Gesellschaft für Geriatrie lassen sich Vorgehensweisen für den deutschen Kontext ableiten. Hier stellt der Algorithmus Stopping Elderly Accidents, Deaths and Injuries (STEADI) der Centers for Disease Control and Prevention (CDC) Screeningmöglichkeiten für PatientInnen ab 65 Jahre dar. Risikogruppen werden mittels eines kurzen Fragenkatalogs identifiziert und Interventionsprogrammen zugeführt (Abb. 3; [3, 29]). Eine Reihe von Cochrane Reviews und systematischen Übersichtsarbeiten belegt die Wirksamkeit multifaktorieller Interventionsprogramme [11, 13–15]. Analog hierzu empfiehlt der STEADI-Algorithmus auf das Sturzrisikoprofil abgestimmte Maßnahmenkombinationen [26]. Hierzu zählen neben Physiotherapie und Medikamentenreview auch Maßnahmen der ergotherapeutischen Wohnraumanalyse und -anpassung. Diese stellt im deutschen Kontext eine Möglichkeit der Evaluation extrinsischer und verhaltensbasierter Sturzrisikofaktoren innerhalb der etablierten ambulanten Versorgung dar.

## Organisatorischer Rahmen der Heilmittelverordnung

Die ärztliche Heilmittelverordnung Ergotherapie ist im ambulanten Sektor in der Heilmittelrichtlinie und den Rahmenempfehlung des GKV-Spitzenverbands geregelt (§ 125, § 92 SGB V). Hiernach umfassen ergotherapeutische Maßnahmen auch die sog. Umfeldberatung (§ 35 Abs. 3 Heilmittel-Richtlinie). Wird eine Heilmittelverordnung ausgestellt, obliegt es dem/der ErgotherapeutIn, eine Umfeldberatung im häuslichen Umfeld zu initiieren. Diese kann unabhängig davon, ob ein Hausbesuch verordnet wurde, einmalig pro Behandlungsfall erfolgen. Bei PatientInnen mit langfristigem Heilmittelbedarf kann sie zusätzlich einmal pro Quartal erbracht werden [12]. Praktische Hinweise zum Ausstellen der Heilmittelverordnung finden sich im Zusatzmaterial online: Anhang 1. Die 120-minütige Umfeldberatung beinhaltet die Analyse des individuellen Umfelds der PatientInnen, die Durchführung von Assessments sowie Empfehlungen zur Wohnraumanpassung, die PatientInnen und VerordnerInnen zur Verfügung gestellt werden. Wohnraumanalysen sind somit innerhalb des bestehenden Systems der Heilmittelerbringung möglich und können einen kosteneffektiven Beitrag zur Sturzprävention leisten.

## Ergotherapeutische Wohnraumanalyse

Durch ErgotherapeutInnen systematisch durchgeführte Wohnraumanpassungen sind mit einer hohen Adhärenz sowie einer Reduktion der Sturzraten assoziiert [2, 4, 30]. Gillespie et al. konnten in einem Cochrane Review zeigen, dass ergotherapeutische Wohnraumanpassungen eine Reduktion der Sturzinzidenz sowie der Anzahl der gestürzten PatientInnen zur Folge hatten [11]. Ebenso ist eine Reduktion der sturzbedingten Verletzungen um 26 % infolge häuslicher Modifikationen beschrieben [19].

**Figure 4:**
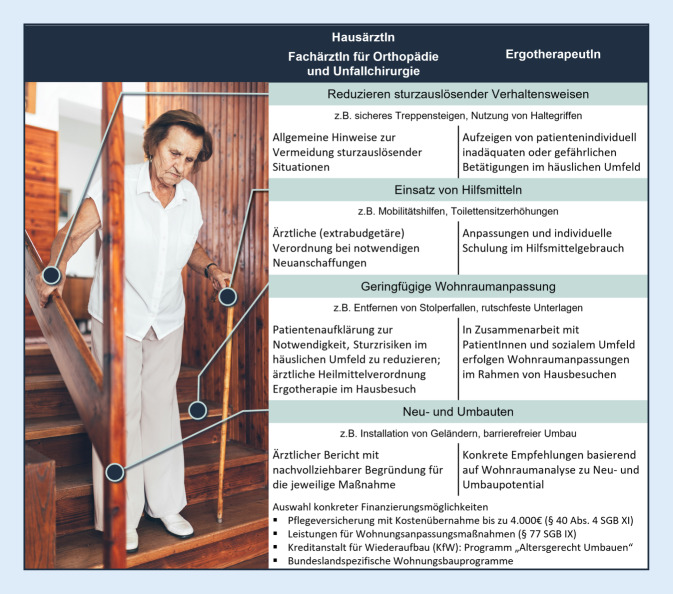

Im Rahmen der ergotherapeutischen Wohnraumanalyse kommen Befundinstrumente zum Einsatz, welche intrinsische, extrinsische und verhaltensbasierte Risikofaktoren für Stürze in der Häuslichkeit der PatientInnen evaluieren. Eine Auswahl ergotherapeutischer Assessments zur Wohnraumanalyse, adressierte Sturzrisikofaktoren und Studien zu psychometrischen Eigenschaften finden sich im Zusatzmaterial online: Anhang 2. Zurzeit gibt es weder international noch für den deutschen Sprachraum einheitlich verwendete Assessments zur Wohnraumanalyse [1]. Es ist daher darauf zu achten, dass ErgotherapeutInnen Assessments verwenden, welche die Evaluation relevanter Sturzrisikofaktoren gewährleisten und psychometrisch getestet sind. Die Ergotherapieleitlinie zur Wohnraumanpassung des amerikanischen Berufsverbands bietet sowohl eine Assessmentübersicht als auch Empfehlungen für den Interventionsprozess [33]. Die in der Leitlinie empfohlenen Interventionsmöglichkeiten zur Wohnraumanpassung finden sich in Abb. 4.

Das Hausbesuchssetting erlaubt es ErgotherapeutInnen, patientenzentriert vorzugehen [33]. Nikolaus und Bach [27] konnten zeigen, dass PatientInnen oft sehr individuelle Methoden nutzen, um sich sicher zu Hause fortzubewegen. Maßnahmen, die ausschließlich auf Edukation und allgemeinen Informationen beruhen, sind daher kaum effektiv [35]. Im Sinne der Sturzprävention sind spezifische Beratungen, Assessments vor Ort, Betätigungsanalysen und gemeinsame Entscheidungsfindung Schlüsselpunkte, um langfristige Adhärenz zu gewährleisten [33].

## Ergotherapeutischer Bericht und Maßnahmen der Wohnraumanpassung

Die gesetzlichen Rahmenbedingungen ermöglichen mit der Wohnraumanalyse ein erstes Assessment des Wohnumfeldes und eine ergotherapeutische Beratung. Empfohlene Maßnahmen können dem/der VerordnerIn in Form eines Kurzberichtes mitgeteilt werden, sofern dies auf der Verordnung vermerkt wurde (Zusatzmaterial online: Anhang 3). Die Studienlage legt nahe, dass Analyse und Empfehlungen allein noch nicht zur Reduktion der Sturzrate beitragen [5]. Die Erkenntnisse der ergotherapeutischen Wohnraumanalyse fließen daher in ein interdisziplinäres Behandlungskonzept ein, um Maßnahmen zur Reduktion der patientenspezifischen verhaltensbasierten und extrinsischen Sturzrisikofaktoren einzuleiten.

Stürze und Sturzursachen werden in ärztlicher Anamnese eruiert und unter Beachtung des ergotherapeutischen Berichts konkrete Verhaltensänderungen zur Vermeidung sturzauslösender Situationen besprochen. Des Weiteren können die empfohlenen Hilfsmittel ärztlich verordnet und deren Anwendung im Rahmen der Ergotherapie beübt werden. Der multimodale Interventionsansatz umfasst zudem das Schaffen einer sicheren Wohnumgebung durch geringgradige Maßnahmen zur Eliminierung von Gefahrenquellen, deren Relevanz durch ärztliche Aufklärung Nachdruck verliehen werden kann. Finanzierungsmöglichkeiten für barrierefreie Neu- und Umbauten sind vielfältig und bedürfen oftmals eines ärztlichen Berichtes, um für die PatientInnen zugänglich zu sein (Abb. 4).

## Diskussion

„It takes a child one year to acquire independent movement and ten years to acquire independent mobility. An old person can lose both in a day.“ (Isaacs [16])

Sturzprävention ist eine multiprofessionelle Aufgabe, die die Gesundheitsfachberufe Ergotherapie, Physiotherapie, Podologie, Pflege, HausärztInnen und weitere FachärztInnen einschließt. Die einzelnen Berufsgruppen haben unterschiedliche Interventionsansätze und Aufgabengebiete zur Prävention von Stürzen. Um die höchste Kosteneffektivität zu erreichen, sollten sich Präventionsprogramme auf Risikogruppen konzentrieren und die Präventionsbemühungen sowohl intrinsische als auch extrinsische und verhaltensbasierte Modifikationen beinhalten [13, 14, 35]. Dabei können intrinsische Sturzrisikofaktoren durch behandelnde ÄrztInnen identifiziert und für die Evaluation extrinsischer und verhaltensbasierter Faktoren kann auf die Ergotherapie zurückgegriffen werden [25].

Für stark sturzgefährdete und bereits gestürzte Personen, solche die Seheinschränkungen oder andere funktionelle Beeinträchtigungen haben, zeigen die ergotherapeutische Wohnraumanalyse und -anpassung eine deutliche Reduktion von Sturzrisiko und Sturzrate [11, 30, 36]. Anzumerken ist, dass eine randomisierte kontrollierte Studie mit ökonomischer Evaluation eine einmalige Wohnraumanalyse für Personen über 65 Jahre ohne gesundheitliche Einschränkungen im Vergleich zur Kontrollgruppe keine Sturzratenreduktion zeigte [5]. Anhand des STEADI-Screenings lassen sich Risikogruppen, welche von ergotherapeutischen Wohnraumanalysen und -anpassungen profitieren, effektiv eingrenzen. Entsprechend konnte im deutschen Kontext für Personen ab 80 Jahren mit ambulantem Pflegebedarf eine deutliche Kosteneffektivität von Wohnraumanpassungen zur Vermeidung von Hüftfrakturen beobachtet werden [21]. Bedeutende Grundlage der Effektivität der ergotherapeutischen Intervention ist daher die Fokussierung auf eine geeignete Zielgruppe.

Darüber hinaus ermöglicht ein verstärkter interdisziplinärer Diskurs mit flächendeckender Einbindung von PatientInnen, Angehörigen und Gesundheitspersonal die notwendige Sensibilisierung, um sturzassoziierte Kosten für das Gesundheitssystem zu minimieren und selbstbestimmtes Altern in der Häuslichkeit zu unterstützen.

## Fazit für die Praxis

- Jährliche Sturzgefährdungsanalysen für PatientInnen ab 65 Jahren können durch HausärztInnen, OrthopädInnen und UnfallchirurgInnen erfolgen.
- Im Rahmen der Verhältnisprävention ermöglichen ergotherapeutische Wohnraumanalysen die Evaluation extrinsischer und verhaltensbasierter Sturzrisikofaktoren.
- Wohnraumanalysen, -beratungen und -anpassungen durch ErgotherapeutInnen sind effektive Maßnahmen zur Reduktion des Sturzrisikos und sind im Rahmen der bestehenden gesetzlichen Rahmenbedingungen umsetzbar.

## Supplementary Information




