# Supplementary material for: Fall prevention in old people through occupational therapy home assessment, consultation and modification: a process outline
Source: Z Gerontol Geriatr. 2022 Sep 7;56(5):408–14. [Article in German] doi: 10.1007/s00391-022-02103-w (PMC10406676; doi:10.1007/s00391-022-02103-w)
Supplement: Supplementary file 1 [file 391_2022_2103_MOESM1_ESM.docx]

# Anhang 1

Beispielhafte Heilmittelverordnung zur ergotherapeutischen Wohnraumanalyse

Ein Hausbesuch wird verordnet, wenn medizinische Gründe den Praxisbesuch verhindern, oder wenn die Therapie im häuslichen Umfeld aus medizinischen Gründen notwendig ist. Die Wohnraumanalyse kann unabhängig von der Verordnung des Hausbesuchs erfolgen.

Diagnosegruppe muss passend zur Diagnose ausgewählt werden

Angabe des Heilmittels „**Ergotherapie**”

Ein entsprechender **Vermerk** informiert die/den ErgotherapeutIn über gewünschte Maßnahmen.


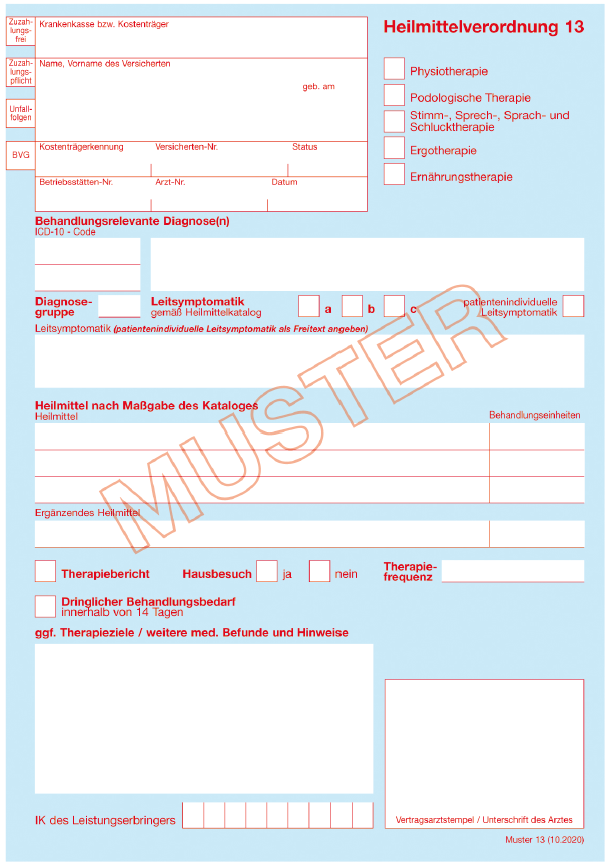


X

Akute Sturzgefährdung

Verhinderung von Stürzen, Wohnraumberatung / Wohnraumanpassung

X

X

X

Mit dem Ankreuzen des Feldes „**Therapiebericht**" erhalten die Verordnenden eine Kurzmitteilung über die erfolgten Maßnahmen.

Sonstige Osteoporose mit pathologischer Fraktur: Sonstige [Wirbelsäule]

M80.88

SB1

Grau hinterlegt: Diagnosebeispiel zur Verordnung im Rahmen des DMP Osteoporose

Die **Leitsymptomatik** entsprechend Heilmittelkatalog (hier SB1 a – Schädigung der Wirbelsäulen- und Gelenksfunktion: Haltung und Haltungskontrolle) kann durch eine patientenindividuelle Leitsymptomatik ergänzt werden.

# Anhang 2 Assessments zur ergotherapeutischen Wohnraumanalyse

**Tab. 1** Ergotherapeutische Assessments zur Wohnraumanalyse, verfügbare Übersetzungen, Durchführungsmodi und psychometrische Eigenschaften.

| Name | AutorInnen | Entwickelt im Fachbereich | Sprache | | Adressierte Sturzrisikofaktoren | | | | Durchführung | | | | | Referenzen Validierung |
| --- | --- | --- | --- | --- | --- | --- | --- | --- | --- | --- | --- | --- | --- | --- |
|  |  |  | Deutsch | Englisch | Intern | Extern | Verhaltens-bezogen | Sonstige | Haus-begehung | Interview | Selbst-einschätzung | ADL Beobachtung | Dauer (min) |  |
| A Way To Stay | SCOPE Access Home Modifications Australia (o.J.) [23] | interdisziplinär |  | ✓ | ✓ | ✓ | ✓ |  | ✓ | ✓ | ✓ |  | o.A. | - |
| Cougar Home Safety Assessment | Fisher & Coolbaugh (2005) [10] | Ergotherapie |  | ✓ |  | ✓ |  | Häusliche Sicherheit | ✓ | ✓ |  |  | 45-60 | [8, 9] |
| Falls Efficacy Scale | Yardley, Beyer, Hauer et al (2005) [28] | Medizin | ✓ | ✓ | ✓ |  |  | Sturzangst |  | ✓ | ✓ |  | 10-15 | [6, 16] |
| Home Assessment Profile (HAP) | Chandler, Duncan, Weiner, Studenski (2001) [2] | Physiotherapie |  | ✓ |  | ✓ |  |  | ✓ |  |  |  | 60-120 | [2] |
| Home Environment Survey (HES) | Rodriguez, Baughman, Sattin et al (1995) [22] | Pflege |  | ✓ |  | ✓ |  |  |  |  | ✓ |  | o.A. | [20] |
| Home Falls and Accidents Screening Tool (Home-FAST) | Mackenzie (2012) [18] | Ergotherapie |  | ✓ |  | ✓ | ✓ |  | ✓ |  |  | ✓ | o.A. | [19, 27] |
| Home Safety Self-Assessment Tool (HSSAT) | Tomita (2017) [25] | Ergotherapie |  | ✓ |  | ✓ |  |  | ✓ |  |  |  | o.A. | [26] |
| Home Screen Scale (HSS) | Johnson, Cusick, Chang (2001) [14] | Pflege |  | ✓ |  | ✓ | ✓ |  |  |  | ✓ |  | o.A. | - |
| Housing Enabler | Iwarsson (1999) [11] | Ergotherapie |  | ✓ | ✓ | ✓ |  |  | ✓ | ✓ |  | ✓ | o.A. | [12, 13] |
| In-Home Occupational Performance Evaluation  (I-HOPE) | Stark, Somerville, Morris (2010) [24] | Ergotherapie |  | ✓ |  | ✓ | ✓ |  | ✓ | ✓ | ✓ | ✓ | 45-60 | [15, 24] |
| Protokoll und Checkliste zur Sturz-Prävention | ErgotherapeutInnenverband Schweiz (2015) [7] | Ergotherapie | ✓ |  |  | ✓ | ✓ |  | ✓ | ✓ |  | ✓ | 60-120 | - |
| Safe at Home Checklist | American Occupational Therapy Association (o.J.) [1] | Ergotherapie |  | ✓ |  | ✓ |  |  | ✓ |  |  |  | 45-60 | - |
| Safer-Home | Oliver, Blathwayt, Brackley, Tamaki (1993) [21] | Ergotherapie |  | ✓ | ✓ | ✓ | ✓ |  | ✓ | ✓ |  | ✓ | o.A. | [3, 17] |
| Westmead Home Safety Assessment (WEHSA) | Clemson, Fitzgerald, Heard (1999) [4] | Ergotherapie |  | ✓ |  | ✓ |  |  | ✓ | ✓ |  |  | Ca. 60 | [4, 5] |
| ADL, Activities of daily life; o.A., ohne Angabe; o.J., ohne Jahr | | | | | | | | | | | | | | |

# Anhang 3


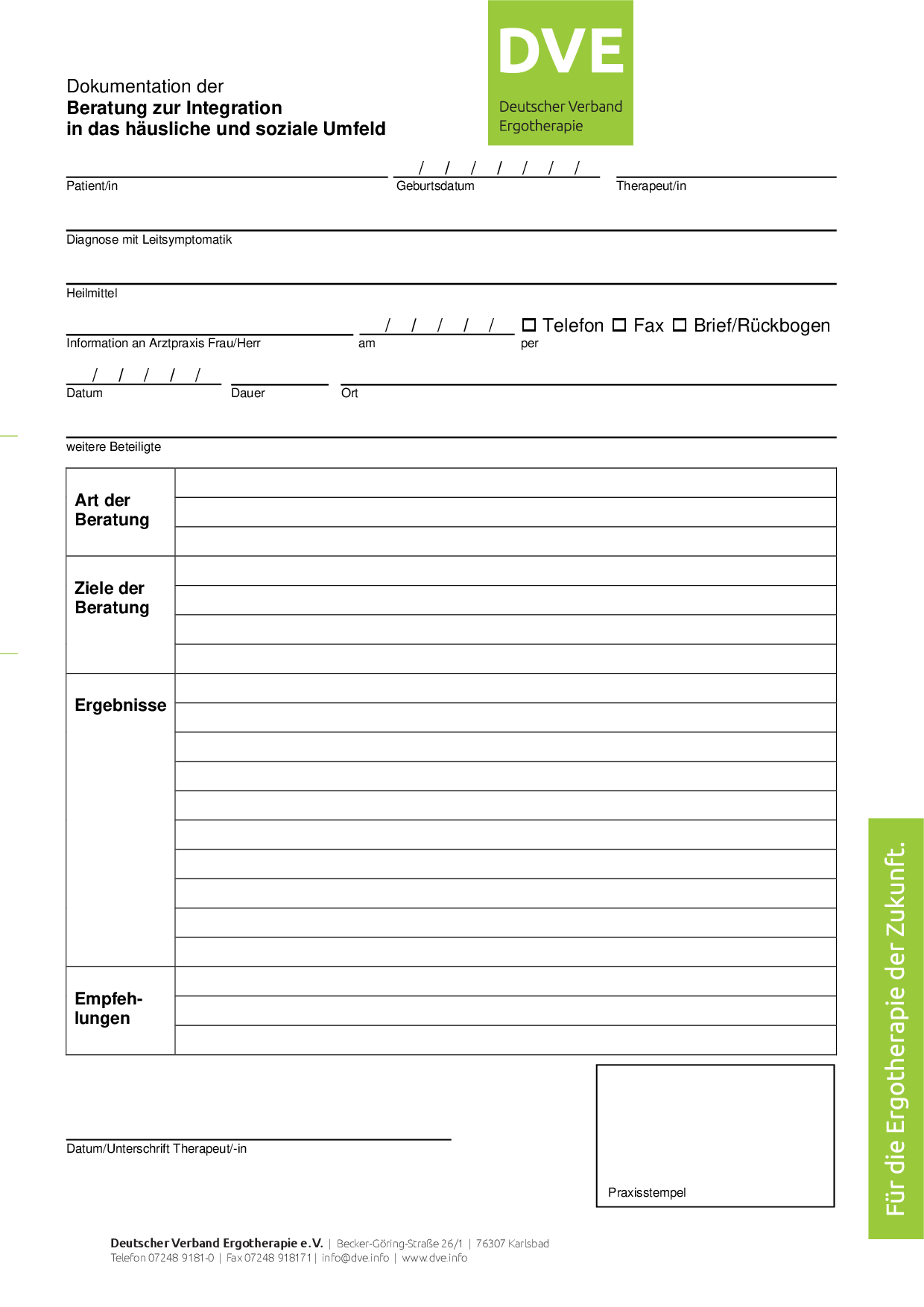
Musterbericht zur ergotherapeutischen Wohnraumanalyse. Mit freundlicher Genehmigung des Deutschen Verbandes Ergotherapie e.V. (DVE), 2021

# Literatur

1. American Occupational Therapy Association, Administration on Aging Safe AT HOME Checklist. https://www.aota.org/-/media/Corporate/Files/Practice/Aging/rebuilding-together/RT-Aging-in-Place-Safe-at-Home-Checklist.pdf. Zugegriffen: 01. Dezember 2021

2. Chandler JM, Duncan PW, Weiner DK, Studenski SA (2001) The Home Assessment Profile—A reliable and valid assessment tool. Top Geriatr Rehabil 16:77–88. DOI 10.1097/00013614-200103000-00010

3. Chiu T, Oliver R (2006) Factor analysis and construct validity of the SAFER-HOME. OTJR Occup Particip Health 26:132–142. DOI 10.1177/153944920602600403

4. Clemson L, Fitzgerald MH, Heard R (1999) Content validity of an assessment tool to identify home fall hazards: The Westmead Home Safety Assessment. Br J Occup Ther 62:171–179. DOI 10.1177/030802269906200407

5. Clemson L, Fitzgerald MH, Heard R, Cumming RG (1999) Inter-Rater Reliability of a Home Fall Hazards Assessment Tool. Occup Ther J Res 19:83–98. DOI 10.1177/153944929901900201

6. Delbaere K, Close JCT, Mikolaizak AS et al (2010) The Falls Efficacy Scale International (FES-I). A comprehensive longitudinal validation study. Age Ageing 39:210–216. DOI 10.1093/ageing/afp225

7. ErgotherapeutInnenverband Schweiz (2015) Wohnraumabklärung zur Sturzprävention in Privathaushalten. Beratungsstelle für Unfallverhütung

8. Fisher G, Burgess B, DiMassimo H et al (2019) Cougar Home Safety Assessment 5.0. Miserocordia University, Dallas

9. Fisher G, Burgess R, DiMassimo H et al (2020) A Best-Practice Modification of the Cougar Home Safety Assessment (CHSA). Am J Occup Ther 74:7411500042p1-7411500242p1. DOI 10.5014/ajot.2020.74S1-PO6405

10. Fisher GS, Coolbaugh K (2005) Development of an environmental safety assessment tool for older persons: Field testing of the Cougar Home Safety Assessments versions 1 and 2. Alzheimers Dement Supplement:63–64. DOI 10.1016/j.jalz.2005.06.233

11. Iwarsson S (1999) The Housing Enabler: An objective tool for assessing accessibility. Br J Occup Ther 62:491–497. DOI 10.1177/030802269906201104

12. Iwarsson S, Nygren C, Slaug B (2005) Cross-national and multi-professional inter-rater reliability of the Housing Enabler. Scand J Occup Ther 12:29–39. DOI 10.1080/11038120510027144

13. Iwarsson S, Slaug B, Fänge AM (2012) The Housing Enabler Screening Tool: Feasibility and Interrater Agreement in a Real Estate Company Practice Context. J Appl Gerontol 31:641–660. DOI 10.1177/0733464810397354

14. Johnson M, Cusick A, Chang S (2001) Home‐Screen: A short scale to measure fall risk in the home. Public Health Nurs 18:169–177. DOI 10.1046/j.1525-1446.2001.00169.x

15. Keglovits M, Somerville E, Stark S (2015) In-Home Occupational Performance Evaluation for Providing Assistance (I–HOPE Assist): An Assessment for Informal Caregivers. Am J Occup Ther 69:6905290010p1-6905290010p9. DOI 10.5014/ajot.2015.015248

16. Kempen GIJM, Todd CJ, Van Haastregt JCM et al (2007) Cross-cultural validation of the Falls Efficacy Scale International (FES-I) in older people: Results from Germany, the Netherlands and the UK were satisfactory. Disabil Rehabil 29:155–162. DOI 10.1080/09638280600747637

17. Letts L, Scott S, Burtney J et al (1998) The Reliability and Validity of the Safety Assessment of Function and the Environment for Rehabilitation (SAFER Tool). Br J Occup Ther 61:127–132. DOI 10.1177/030802269806100309

18. Mackenzie L (2012) Home Falls and Accidents Screening Tool (Home-FAST). University of Sydney, Sydney

19. Mackenzie L, Byles J, Higginbotham N (2000) Designing the Home Falls and Accidents Screening Tool (HOME FAST): Selecting the Items. Br J Occup Ther 63:260–269. DOI 10.1177/030802260006300604

20. Morgan R, Devito C, Stevens J et al (2005) A self-assessment tool was reliable in identifying hazards in the homes of elders. J Clin Epidemiol 58:1252.e1-1252.e21. DOI 10.1016/j.jclinepi.2005.04.001

21. Oliver R, Blathwayt J, Brackley C, Tamaki T (1993) Development of the Safety Assessment of Function and the Environment for Rehabilitation (SAFER) Tool. Can J Occup Ther 60:78–82. DOI 10.1177/000841749306000204

22. Rodriguez JG, Baughman AL, Sattin RW et al (1995) A standardized instrument to assess hazards for falls in the home of older persons. Accid Anal Prev 27:625–631. DOI 10.1016/0001-4575(95)00016-S

23. SCOPE Access Home Modifications, DisabilityCare Australia’s National Disability Insurance Scheme (NDIS) A way to stay - Home Modification Assessment and Planning Tool. http://www.scopehomeaccess.com.au/wp-content/uploads/2015/07/AWTS.pdf. Zugegriffen: 23. September 2021

24. Stark SL, Somerville EK, Morris JC (2010) In-Home Occupational Performance Evaluation (I–HOPE). Am J Occup Ther 64:580–589. DOI 10.5014/ajot.2010.08065

25. Tomita M (2017) Home Safety Self Assessment Tool (HSSAT) v.5. Rehabilitation Department, University Buffalo

26. Tomita MR, Saharan S, Rajendran S et al (2014) Psychometrics of the Home Safety Self-Assessment Tool (HSSAT) to Prevent Falls in Community-Dwelling Older Adults. Am J Occup Ther 68:711–718. DOI 10.5014/ajot.2014.010801

27. Vu T-V, Mackenzie L (2012) The inter-rater and test–retest reliability of the Home Falls and Accidents Screening Tool. Aust Occup Ther J 59:235–242. DOI 10.1111/j.1440-1630.2012.01012.x

28. Yardley L, Beyer N, Hauer K et al (2005) Development and initial validation of the Falls Efficacy Scale-International (FES-I). Age Ageing 34:614–619. DOI 10.1093/ageing/afi196
